# Recent advances in mechanisms ensuring the pairing, synapsis and segregation of XY chromosomes in mice and humans

**DOI:** 10.1007/s00018-024-05216-0

**Published:** 2024-04-23

**Authors:** Matteo Lampitto, Marco Barchi

**Affiliations:** 1 Section of Anatomy, Department of Biomedicine and Prevention, University of Rome Tor Vergata, Rome, Italy; 2 Section of Anatomy, Department of Medicine, Saint Camillus International University of Health Sciences, Rome, Italy

**Keywords:** SPO11β, SPO11α, PAR, Sex chromosomes, XY, Meiotic recombination, Meiosis, Aneuploidy, Chromosome structure, Double strand breaks (DSBs), Klinefelter syndrome, Turner syndrome

## Abstract

Sex chromosome aneuploidies are among the most common variations in human whole chromosome copy numbers, with an estimated prevalence in the general population of 1:400 to 1:1400 live births. Unlike whole-chromosome aneuploidies of autosomes, those of sex chromosomes, such as the 47, XXY aneuploidy that causes Klinefelter Syndrome (KS), often originate from the paternal side, caused by a lack of crossover (CO) formation between the X and Y chromosomes. COs must form between all chromosome pairs to pass meiotic checkpoints and are the product of meiotic recombination that occurs between homologous sequences of parental chromosomes. Recombination between male sex chromosomes is more challenging compared to both autosomes and sex chromosomes in females, as it is restricted within a short region of homology between X and Y, called the pseudo-autosomal region (PAR). However, in normal individuals, CO formation occurs in PAR with a higher frequency than in any other region, indicating the presence of mechanisms that promote the initiation and processing of recombination in each meiotic division. In recent years, research has made great strides in identifying genes and mechanisms that facilitate CO formation in the PAR. Here, we outline the most recent and relevant findings in this field. XY chromosome aneuploidy in humans has broad-reaching effects, contributing significantly also to Turner syndrome, spontaneous abortions, oligospermia, and even infertility. Thus, in the years to come, the identification of genes and mechanisms beyond XY aneuploidy is expected to have an impact on the genetic counseling of a wide number of families and adults affected by these disorders.

## Introduction to meiosis: key concepts

**Fig. 1:**
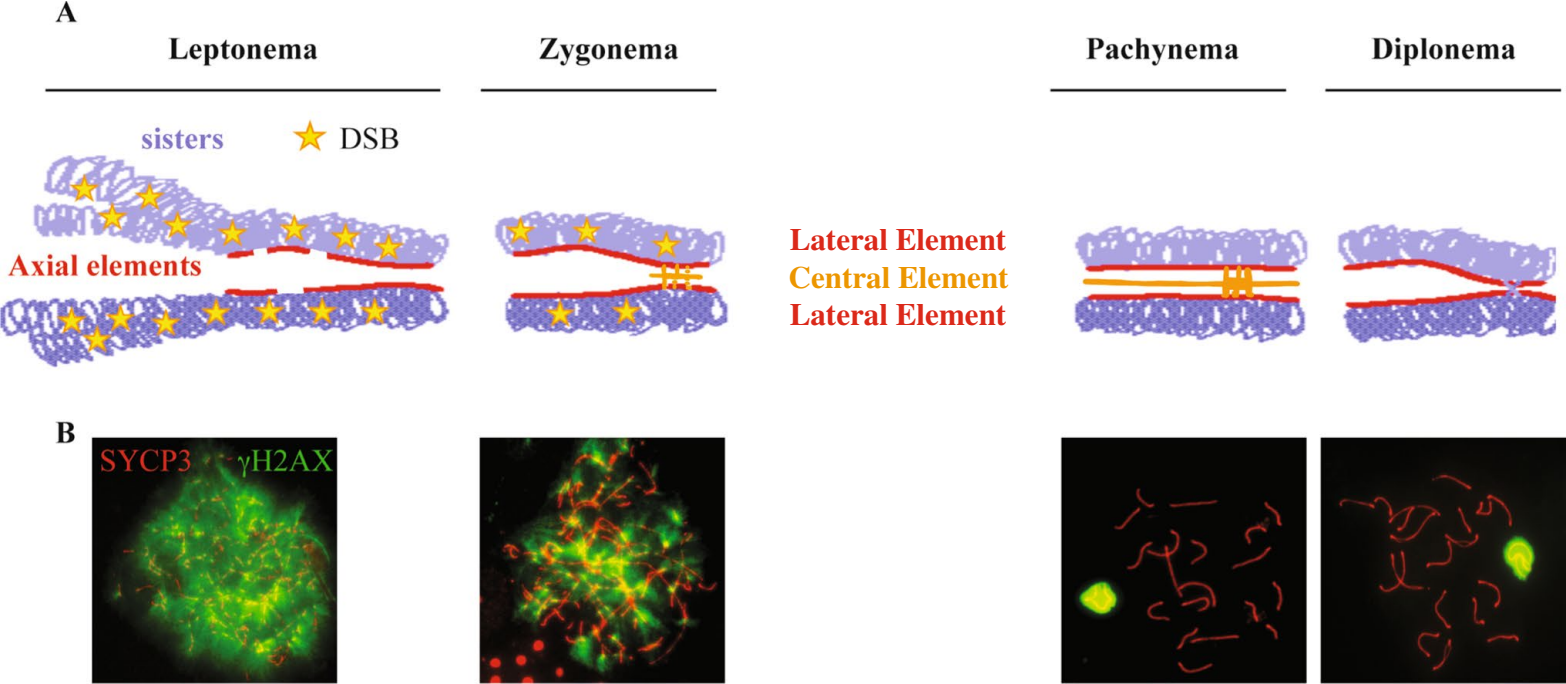
DSB formation and assembly of the SC during meiotic prophase I. **A** In leptonema of normal meiotic prophase, the sister chromatids of each chromosome develop a proteinaceous axis (the SYCP3-positive axial element), with chromatin extending out in loops. Associations form between the axes of homologous chromosomes and extend progressively during the zygotene stage, forming the tripartite Synaptonemal Complex (SC) that comprises both axial elements (red) and transverse filaments of the central element (orange) that connects them. Synapsis is completed by pachynema, so that the SC joins homologous chromosomes along their entire lengths. Meiotic recombination involves the formation and repair of DSBs (stars) that start to be made at leptonema up to zygonema on asynapsed autosomal homologous. **B** In the mouse, formation of the SC and synapsis of the homologous is monitored cytologically on prophase I meiotic chromosomes spreads, by staging them with an anti-SYCP3 antibody (red). DSBs are typically monitored by examining γH2AX [53], a phosphorylated form of histone H2AX, which appears on chromosomes in early meiotic prophase I (i.e., from leptonema to zygonema) in a SPO11-dependent fashion [4, 6]. At pachynema and diplonema γH2AX persists only on the sex chromosomes as the part of the MSCI mechanism [48]

A fundamental property of life is the ability to reproduce. In sexually reproducing higher eukaryotes, the generation of gametes, which are sperm and eggs, occurs through meiosis. Meiosis is a biological process in which germ cells, after a round of DNA replication, divide twice, halving DNA content. Meiosis not only grants the re-establishment of diploidy of the embryo after fertilization, but also allows for exchange of genetic material between maternal and paternal chromosomes. This genomic shuffling creates new genetic combinations that ultimately contribute to genetic diversity, leading to the creation of increasingly robust or specialized offspring. The key biological mechanism at the base of genetic reassortment is the homologous recombination (HR) of repair of DNA double strand breaks (DSBs), which are physiologically introduced into the genome during meiotic prophase I (Fig. 1A-B). Unlike mitosis, meiotic HR preferentially utilizes the homologous chromosome over the sister chromatid, as template for DSB repair. This may result in reciprocal exchange of genetic material between regions with strong similarity, forming crossovers (COs) [1]. In mice, DSBs are generated by SPO11 (the ortholog of subunit A of TopoVI DNA topoisomerase) [2–7] in complex with the TopoVI B-like subunit (TOPOVIBL) [7]. The *Spo11* gene is conserved in humans [2, 5] and single nucleotide polymorphisms of *SPO11* are associated with male infertility and decreased ovarian reserve [8–11]. Key TOPOVIBL domains are also conserved in humans [7], suggesting a shared mechanism for the formation of DSBs between species. In mouse spermatocytes, DSBs start to be made at leptonema, peak at zygonema, and decrease in number as cell progresses to pachynema. DSBs are distributed along the entire length of homologous autosomes (the homologous), each formed by two sister chromatids. Conversely, given that mouse (and human) sex chromosomes are heteromorphic, DSB formation and recombination is restricted within a short region of homology between them, the PAR, which genetically behave like autosomes. Mice have a single PAR (mPAR) [12], while human sex chromosomes have two PARs: PAR1 and PAR2. PAR1 corresponds to mPAR, as CO occurs only rarely in PAR2 [13].

In autosomes, only about 10–25% of DSBs is repaired by HR with the formation of COs [14–16]. COs produce new combinations of DNA sequences, resulting in enhanced genetic variation. The remaining DSBs are repaired by interhomolog recombination without reciprocal exchange, resulting in the formation of non-crossover (NCO) products [1, 15, 17, 18]. The latter, by allowing transmission distortion of genetic information and mutations, are also considered to provide a significant contribution to genome evolution [15, 19], and are essential to guarantee DSB-driven alignment and synapsis of the homologous (see below). Repair of DSBs with the formation of a CO occurs with reciprocal DNA exchange between parental chromosomes [15, 20]. By doing so, in addition to shuffling the genome, these events lead to the formation of interhomolog DNA links that are cytologically identifiable as chiasmata [21–23]. Chiasmata, by physically linking homologous, play a key role in the co-orientation of the sisters of each homolog to opposite spindle poles [24], counteracting forces exerted by the centromere–attached microtubules. This ensures proper alignment of homologous at the metaphase I spindle and segregation in daughter cells. If CO formation fails, meiotic chromosomes segregate randomly, with consequent meiotic arrest and infertility due to activation of the Spindle Assembly Checkpoint (SAC) mechanisms of selection [25, 26]. Hence, each homologous pair (bivalent) requires at least one CO for chromosomes to segregate properly [20], known as the “obligatory CO”. In male sex chromosomes, formation of the obligatory CO is restricted to the PAR. A failure in the generation of at least one DSB in the X-PAR and/or Y-PAR results in missegregation of the sex chromosomes. In mice, according to phenotypic penetrance, this can lead to infertility [27] or sub fertility [28], with generation of aneuploid sperm for the sex chromosomes [29, 30]. In men, infertile, oliogozospermic and oligoasthenoteratozoospermic patients manifest significantly higher levels of XY disomy [31–33], probably due to reduced recombination in the PAR [32, 34]. The lack of CO formation between the XY chromosomes also occurs in fathers with progeny of Klinefelter Syndrome (KS) (47, XXY) [35–37], which is the most common (1:500–1:1000 [38]) sex chromosome disorder of paternal origin. Nondisjunction of XY chromosomes also likely cause of fathering a progeny with Turner syndrome (TS).  Therefore, understanding the genetics of XY recombination in mouse and humans is key to understand the etiology of sex chromosome disorders.

## Recombination-independent mechanisms of XY chromosome pairing and synapsis

**Fig. 2:**
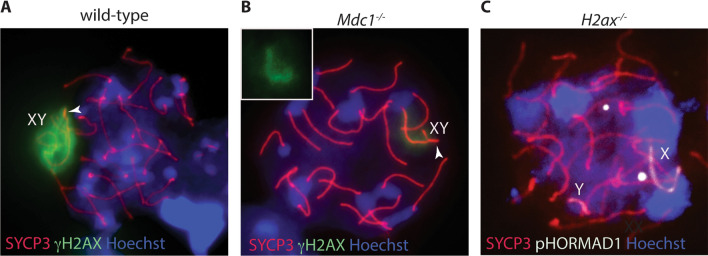
Chromosome spreads of mouse spermatocytes at pachynema. **A** In wild type spermatocytes, the SC axial element SYCP3 (red) extends along fully paired autosomes, while the XY chromosomes only synapse at PAR. The X and Y chromosomes are embedded within the sex body, a chromosome domain identified by the phosphorylation of histone H2ax (γH2AX, green). **B** In *Mdc1*^*−/−*^ spermatocytes sex body formation partially fails, due to inefficient spreading of γH2AX to chromatin loops [49]. XY chromosomes synapse in a large fraction of cells, although with reduced proficiency compared to wild type [51]. **C** In *H2ax*^*−/−*^ spermatocytes, sex body formation fails [50], and the X and Y chromosomes (labelled with the antibody that recognizes the phosphorylation status of the HORMA [Hop1, Rev7 and Mad2] domain one protein [HORMAD1] [164]; white) are unsynapsed with a higher percentage compared to *Mdc1*^*−/−*^ cells [51]. In **A** and **B**, white arrows head point to the PAR

Meiotic prophase I differs substantially from mitotic prophase, not only because of the formation of COs but also because of the process that drives pairing and synapsis of parental chromosomes. Pairing between the homolog pairs (that is, approaching and juxtaposing of the chromosomes) begins after DNA duplication at preleptonema (prophase I), prior to SPO11-mediated DNA cleavage [39]. This recombination-independent mechanism requires an unknown DSB-independent activity of SPO11 and that of SUN1, a nuclear membrane protein that binds telomeres to the nuclear envelope [40]. The occurrence of pairing most likely facilitates the initiation of synapsis [39], that is, stable axial alignment of the homologous, which begins with the formation of DSBs and the assembly of the synaptonemal complex (SC) (see below). Interestingly, a recent study pointed out that homologous pairing at spermatogonia-early preleptotene stage occurs in over 70% of the mouse homologous, including the XY chromosomes. In the latter, pairing frequency further increases by mid-preleptotene stage occurring in over 85% of the cells [41]. This suggests that pairing promotes spatial proximity between the XY chromosomes, as proposed for non-sex chromosomes [39, 41]. However, it is unclear whether this proximity may play a role in the promotion of synapsis between the XY chromosomes, as synapsis does not occur until late zygotene [27]. Furthermore, whether the DSB independent function of SPO11 and that of SUN1 are required for the XY pairing has not been investigated. A key feature of meiotic cells is the assembly of the SC, a tripartite zipper-like proteinaceous structure that mediates homologous chromosome alignment and synapsis during prophase I [42–44]. Under physiological conditions the SC is formed along the full length of autosomes and at the PAR of bivalents that are undergoing recombination (Fig. 1A). Nevertheless, the SC also assembles in short stretches, between non-homologous chromosomes of DSB-deficient mutants, such as *Spo11*^*−/−*^ [4, 6, 45], stabilizing synapsis, locally. Therefore, the SC also promotes recombination-independent synapsis, a function that lies in the ability of SC proteins to undergo self-assembly [46, 47]. An additional accessory mechanism that probably promotes spatial proximity between XY is the formation of the sex body. The sex body is a chromatin domain resulting From Meiotic Sex Chromosome Inactivation (MSCI), the process that silences XY-associated genes of heterologous (asynapsed) XY chromosomes regions at pachynema (see [48] and references therein). A key event in MSCI is phosphorylation of histone H2AX at serine 139 (γH2AX) (Fig. 1B and Fig. 2A), which attracts MDC1 (mediator of DNA damage checkpoint 1), a γH2AX binding partner. MDC1 promotes spreading of γH2AX to chromatin loops (Fig. 2B), for effective silencing of XY associated genes [48, 49]. In absence of *H2ax* X and Y are asynapsed (Fig. 2C) with a higher frequency compared to that of *Mdc1*^*−/−*^ cells, in which the sex body formation fails only partially (Fig. 2B) [49–51]. Therefore, it is likely that nuclear compartmentalization of XY chromosomes elicited by the formation of the sex body plays a role in bringing the XY closer, promoting PARs proximity, pairing and synapsis [51]; possibly suppressing illegitimate recombination of non-homologous regions [52].

## Recombination-dependent mechanism of chromosome pairing and synapsis

In mice carrying the mutation of the catalytic tyrosine of *Spo11* (Y/F substitution), synapsis of the homologous is disrupted, indicating that pairing achieved through recombination–independent mechanisms (see above) is dependent on the DSB activity of SPO11 [39]. In accordance, previous studies have demonstrated that DSB formation precedes synapsis of the homologous [53], and that when the expression level of SPO11 is reduced below a critical threshold, and DSB numbers decrease considerably, homolog synapsis fails, with the consequent elimination of defective spermatocytes by apoptosis [54, 55]. In a simplified view of the events driving the synapse between homologs, formation of DSBs by SPO11 is followed by processing of DSBs into 3’ ssDNA ends, which are required for the search of the complementary sequences in the homologous chromosome, leading to interhomolog interactions pairing and synapsis, with the assembly of the SC [1] (Fig. 1A).

Despite DSB numbers may vary considerably among spermatocytes [15, 56], proper establishment of synapsis between autosomes always requires that DSBs are initiated at multiple sites along chromosome length (Fig. 1A). It is believed that starting homology-dependent DNA interactions from multiple recombination events enforces homolog pairing during meiotic prophase [57], while suppressing interactions between non-allelic homologous sequences [58]. For this reason, the DSB numbers per cell (> 200 on average in mice and humans) [14, 15, 27, 55] substantially exceed those of COs. Under these circumstances, smaller autosomes and sex chromosomes which physiologically receive a low number of DSBs, are particularly vulnerable to DSBs decrement, engaging in non-homologous synapsis [54]. Thus, a robust wave of DSBs at leptonema and zygonema paves the way for the success of meiosis.

### DSB formation in the PAR

Based on the average frequency of DSBs in mouse spermatocytes it is estimated that fewer than one DSB form per ten Megabase pairs [27]. The PAR is ~ 0.7 Megabase long [59, 60]. This would predict that the PAR receives fewer than one DSB for every ten meioses (that is, a > 90% failure rate). In contrast, PAR experiences one or two DSBs per meioses [27, 61, 62]. Therefore, the formation of DSBs in the PAR is 10–20 times higher than the average autosomal region [27]. It follows that there must be mechanisms in place that implement the probability that SPO11 is recruited and active in this region. In addition to the frequency, the timing with which DSBs are formed in autosomes and in the mPAR is also different. In autosomes, DSBs start to form at leptonema, peak in number at zygonema, and decrease by late zygonema and pachynema. Conversely, a DSB is detected in the X-mPAR and Y-mPAR, more frequently at late zygonema [27]. The different timings of DSB formation in autosomes and sex chromosomes correlate, in mice, with the expression of two splicing isoforms of *Spo11*: *Spo11β* and *Spo11α* [2, 5]*. SPO11β* is expressed earlier, by leptonema, when DSB formation starts nucleus-wide on autosomes, whereas *SPO11α* starts to be expressed (concomitantly with *Spo11β*) in late prophase I [2, 5, 63]. These two isoforms differ from each other for the inclusion (*Spo11β*) or skipping (*Spo11α*) of exon2 [2, 5, 63]. The ratio of *Spo11β* and *Spo11α* isoforms is dependent on modulation of RNA polymerase II and the recruitment of splicing factors [64, 65]. Consistent with their timing of expression, it has been proposed that SPO11β is required for DSB formation on autosomes, while SPO11α on the PAR. Using the mouse as a model system, Kauppi et al. demonstrated that transgenic expression of SPO11β in a *Spo11*^*−/−*^ genetic background (*Spo11β*-only transgenic mouse), complements autosomal synapsis defects observed in *Spo11*^*−/−*^ mice [4, 6], while XY chromosomes remain asynapsed, with consequent infertility [27]. However, a subsequent study using the same animal model showed that defective XY synapsis can be reduced to 50% in mice with a different genetic background, and males are fertile [28]. This indicates that, other than SPO11β, the expression of other *Spo11* splice isoforms is not essential for male fertility. This calls into question what the function of SPO11α is, and why genetic background changes impact the proficiency of XY recombination and synapsis. Using a knock-in mouse model that expresses only SPO11β under its physiological promoter (*Spo11βki*-only mouse) we confirmed that DSB frequency in the PAR changes with genetic background. Moreover, we observed that it fails more frequently in the Y-PAR than the X-PAR [30]. Furthermore, we demonstrated using the *Spo11αki* mouse model, that concomitant expression of SPO11α with SPO11β strongly boost formation of DSBs in the Y-PAR, regardless of the genetic background [30]. Therefore, it can be concluded that the expression of the single SPO11β isoform increases the risk of XY asynapsis. To date, the exact molecular mechanism behind the cooperation of *Spo11* splice isoforms remains unknown. According to in vitro protein–protein interaction experiments, SPO11α is unable to interact with TOPOVIBL [7]. Thus, it is assumed that in vivo, SPO11α cannot form heterotetramers with SPO11β and TOPOVIBL. It has been hypothesized that SPO11α may titrate an inhibitor of TOPOVIBL, for example via a protein–protein interaction, raising the possibility that the heterotetramers formed by SPO11β and TOPOVIBL [7] form the DSB on PAR, in late zygonema [66]. However, this remains speculation.

#### Epigenetic determination of DSBs hotspot

Mammalian meiotic DSBs are not randomly distributed but they occur preferentially in genomic regions called “hotspots”, which are positioned by the (widely conserved) mouse meiosis-specific methyltransferase PRDM9 (PR domain-containing 9) protein [67–69]. PRDM9 is a zinc finger protein that, through interaction with the HELLS chromatin remodeler binds DNA [70, 71] and trimethylates histone 3 in Lysine-4 (H3K4me3) and Lysine-36 (H3K36me3) in nearby nucleosomes [72–74], providing access to the DSB initiating complex in nucleosome-depleted regions [68, 69, 73, 75–77]. Meiotic DSBs form in normal numbers in mouse spermatocytes with inactivated *Prdm9*, but occur at “default sites”, which are PRDM9-independent H3K4me3 enriched regions (such as promoters and enhancers) that are rarely targeted in wild type mice [75, 76]. This relocation parallels a defect in the repair of DSBs, with a consequent failure of synapsis between homologues and may result in sterility [75, 76, 78, 79]. Heterozygosity of *Prdm9* also leads to sterility in some hybrid mice [80], while genetic background shift in mice with *Prdm9* loss of function mutation partially restores fertility [78, 81]. These results suggest the presence of genetic modifiers of PRDM9 function, for example the expression, in specific genetic contexts, of other chromatin modifiers partially substituting PRDM9 function, when the gene is deleted. Beyond H3K4me3 and H3K36me3, H3 lysine 9 acetylation (H3K9ac) is also enriched concurrently at recombination autosomal hotspots. H3K9ac also promotes chromatin openness, enabling DSB repair by homologous recombination [82]. On this regard The H3K4me3 and H3K36me3 reader ZCWPW1 (Zinc Finger CW-Type and PWWP Domain Containing 1) is recruited to recombination hotspots by PRDM9 and is essential for the execution of early repair steps at DSBs hotspots, by antagonizing histone deacetylase proteins [82–85]. In mouse, the PAR region contains a large H3K4me3 hotspot [75], which, however, is generated independently of PRDM9 [75]. To date, the methyltransferase responsible for H3K4me3 deposition in the mPAR has not been identified; hence, the function of H3K4me3 in the PAR remains to be experimentally validated. This contrasts with what has been found in humans, where PRDM9 does localize peaks of recombination in the PAR1 [86], and it is thus likely relevant for CO formation, in this region.

#### Trans-acting factors of DSB formation in the PAR: the essential role of ANKRD31

**Fig. 3:**
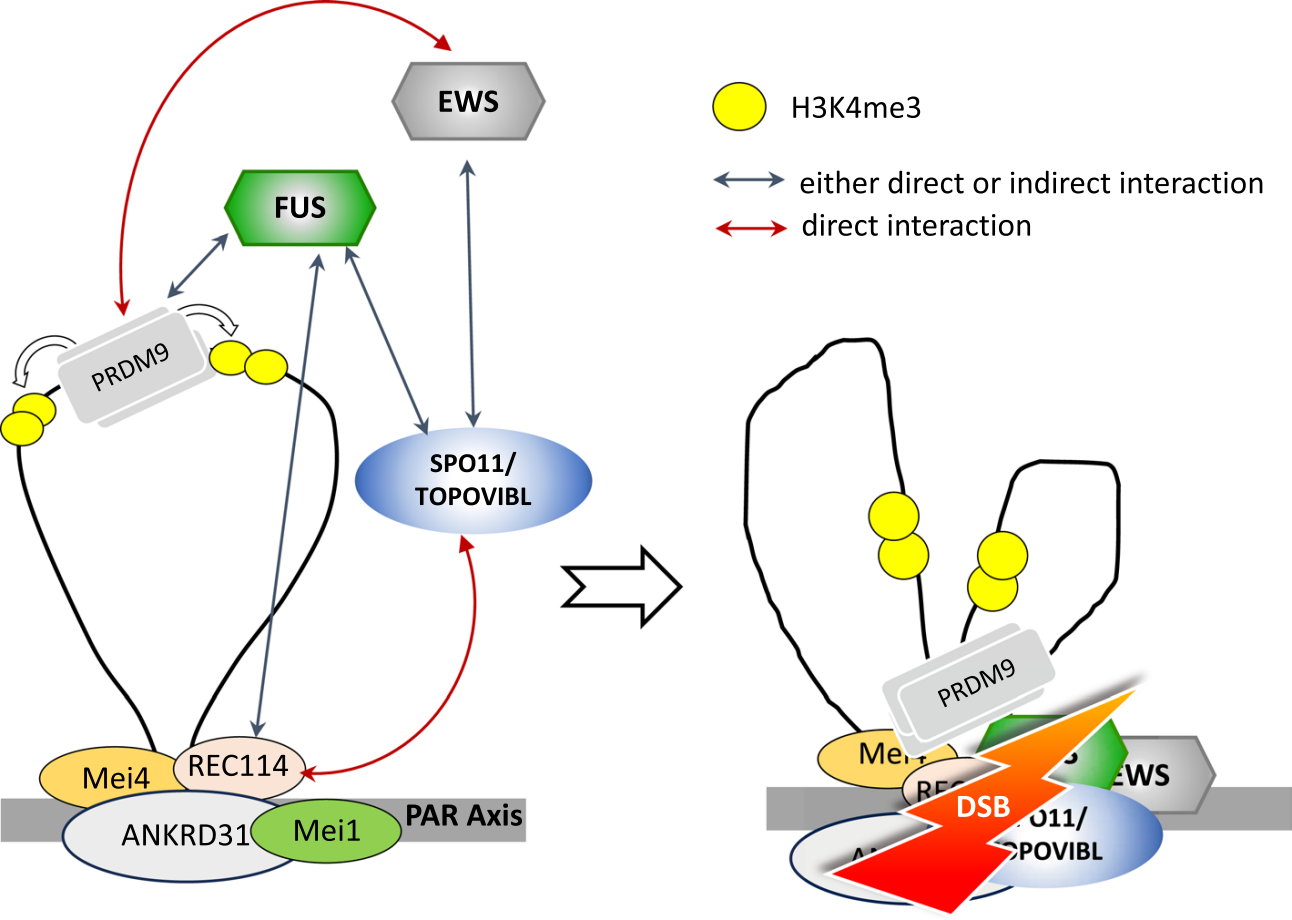
Putative axis loop tethering model of the roles of FUS and EWS1 at the human PAR1 hotspot. FUS-PRDM9 interaction (might be indirect) links H3K4me3 K36me3-marked chromatin loops at the PAR1 with the SPO11 complex. The FUS-REC114 cooperation may enhance the proximity of the PAR1 hotspot with the axis and SPO11 auxiliary proteins. TOPOVIBL interacts directly with REC114 [66], possibly targeting the function of SPO11/TOPOVIBL onto axis. EWS1 may reinforce the role of FUS and TOPOVIBL via its interaction with PRDM9 and/or SPO11 (adapted from [103])

Functional activation of SPO11 at epigenetically marked hotspots requires the expression of several genes encoding auxiliary proteins of SPO11, namely: *Iho1*, *Mei1*, *Mei4 Rec114, Ankrd31* (RMMAI proteins [62, 87–90]), which gene products form aggregates on the chromosome axes (the site where DSBs are made by SPO11) in advance of DSB formation [62, 87, 89, 91, 92]. Interestingly, mouse RMMAI aggregates form onto PAR much larger clusters (RMMAI blobs) than onto autosomes. RMMAI blobs also form onto autosomes at the (non-centromeric) telomeric regions of chromosomes 13, 9 and 4, which (like at the PAR) undergo DSB formation with at delayed timing compared to most autosomal hotspots [62]. Sequence analysis has revealed that the PAR and telomeres of these autosomes share the enrichment of tandem arrays of 31-bp repeats reach region known as mo-2 minisatellite [62]. In the mouse strains where mo-2 copy numbers in telomeres are lower, the aggregation of RMMAI factors (such as REC114) is lower [62]. Therefore, it has been concluded that the mo-2 minisatellite acts as a *cis*-acting determinant for RMMAI hyper aggregation [62]. Although the formation of DSBs in both autosomal and PAR hotspots require RMMAI aggregates, not all components of the complex are equally important for these regions. While expression of MEI1, MEI4 and REC114 is crucial for DSB formation across the entire genome [89], that of IHO1 (which is a direct binding partner of the axis associated protein HORMAD1 [87]), is obligate for autosomal DSB formation, while it is dispensable at the PAR [62, 93]. Conversely, the function of ANKRD31is critical in the PAR and PAR-like autosomal telomeres, and not essential at autosomal hotspots [90, 94]. In *Ankrd31*^*−/−*^ spermatocytes near all of cells fails XY synapsis, and cells with achiasmata sex chromosomes arrest at MI, due to the activation of the SAC, leading to sterility [90, 94]. ANKRD31 is a direct REC114 interacting partner [90, 94]; therefore, it is speculated that ANKRD31 by recognising the *cis*-acting features of the PAR, recruits REC114 and other proteins by direct interaction, promoting DSB formation [94]. In this regard, it has recently been shown that REC114 also interacts directly with MEI1 ([93], Preprint) and TOPOVIBL [66] (Fig. 3). However, the interaction of TOPOVIBL and ANKRD31 with REC114 is mutually exclusive [66]. Therefore, given the essential function of ANKRD31 at the PAR [90, 94], it is not yet clear what is the exact interplay between these factors with TOPOVIB and SPO11. The interaction of ANKRD31 with REC114 is mediated by a conserved C-terminal region of ANKRD31, which wraps around the N-terminal PH domain (Pleckstrin Homology) of REC114, as shown by crystal structure studies [94]. Emphasizing the importance of such interaction, a recent study reported that in mice in which the REC114-ANKRD31 interaction is disrupted due to C-terminal truncation of ANKRD31, DSB formation at the PAR and XY synapsis is abolished, mimicking an *Ankrd31* null phenotype [95]. The authors also demonstrated that when a missense mutation (E to A mutation at aa 1831) is introduced in the C-terminal of mouse ANKRD31, beside the ANKRD31-REC114 interaction was severely biodisrupted in Yeast 2-Hybrid assay, meiotic defects in homozygous mutants were much milder than in mice carrying the C-terminal truncation. This indicates that in vivo, the interaction is at least partially retained; perhaps strengthened by the network of interactions of ANKRD31 with other partners [95]. It is possible that ANKRD31 functions as a scaffold, interacting with multiple proteins at different times. In agreement with this concept, it has also demonstrated that ANKRD31 can also interact directly with MEI1 through one of its Ankyrin repeats [95], and with two more protein factors, namely: ZMYM3 (zinc finger, myeloproliferative, and mental retardation-type 3) and PTIP (Pax transactivation domain interacting protein; also known as PAXIP1) [62]. ZMYM3 is a chromatin interacting protein which promotes HR repair mediated by BRCA1 in somatic cells [96], and its deletion in mouse causes arrest of spermatocytes at MI [97]; which is compatible with a defect in CO formation between autosomes and/or sex chromosomes [25, 27]. PTIP, is an essential component of the activating H3K4me3 complex [98], and it is implicated in DNA damage repair [99]. Deletion of *Ptip* in testis causes arrest of spermatocytes at MI [100]. PTIP is not needed for global maintenance of H3K4me3 status [100], but its function might be required at specific subregions such as the mouse PAR, where the large H3K4me3 hotspot is PRDM9 independent [75]. Importantly, both ZMYM3 and PTIP are enriched at the PAR [62], which is a strong indication of their co-involvement in the formation of DSBs, and/or processing, in this region.

#### Additional trans-acting factors with potential function in the PAR: EWS1 and FUS

The protein EWS1 (Ewing’s sarcoma breakpoint region 1) along with the gene products encoded by FUS (fused in sarcoma)/TLS (translocated in liposarcoma) and the TATA box binding associated factor 15 (*Taf15*), are RNA and DNA binding proteins that belong to the FET (FUS, EWS, TAF15) family of proteins [84]. Previous studies have demonstrated that *Ewsr1*^*−/−*^ spermatocytes are deficient in synapsis of the autosomes and XY chromosomes, indicating their essential function during meiosis [101, 102]. By using the *Spo11βki*-only and *Spo11αki*-only models [30], we demonstrated that EWSR1 co-immunoprecipitates with SPO11β, SPO11α and REC114 [103]. Given the significance of SPO11α in DSB formation at the PAR [30], this observation supports a role for EWS1 in XY recombination. Intriguingly, homolog synapsis is also defective in spermatocytes lacking FUS/TLS expression [104]. FUS also co-immunoprecipitates with SPO11 splice isoforms and with REC114, and it localizes at the PAR hotspot [103]. Therefore, FUS/TLS is also a likely player in XY recombination.

The formation of DSBs occurs in the context of the spatial organisation of meiotic chromosomes, which form chromatin loops that extend from a linear protein axis. Based on the yeast model, which predicts that the DSB machinery assembled on the axis captures and breaks DNA loops, it has been proposed that in mammals, DSB occurs in PRDM9-marked regions on DNA loops, which are next attached to the axis, where DSBs are made and repaired by assembly of DNA repair factors [105, 106]. Both EWSR1 and FUS/TLS co-immunoprecipitates with PRDM9 in spermatocytes [103, 107] Therefore, it is speculated that these protein factors binding PRDM9 on chromatin loops are tethered to the axis (bound REC114) and with SPO11, promoting DSB formation [103]. In mice DSBs form in the PAR independently from PRDM9 [75]. However, in man, PRDM9 does localize at the PAR1 [86], therefore FET family proteins, with same mechanism hypothesized for the autosomes in mice, may facilitate DSB formation on the PAR1 chromosome axis (Fig. 3).

In addition to FET family proteins, in mice, PRDM9 also interacts directly with CXXC1 (CxxC finger protein 1), a H3K4me3 reader ortholog of *S. cerevisiae* Spp1 [108] in mammals [109], CDYL (chromodomain-containing Y chromosome-like) and EHMT2 (euchromatic histone methyltransferase 2). These protein factors are expressed in spermatocytes with the same timing as PRDM9 and are though to facilitate the association of putative hotspot sites in DNA loops with the chromosomal axis ([107], reviewed in [110]). To date, CDYL and EHMT2 function still await experimental validation, in vivo, while the function of CXXC1 at hotspots remains uncertain, as phenotypic characterization of *Cxxc1* null mice with a C57BL/6 genetic background, provided contradictory results, likely due to differences in the experimental settings [109, 111].

#### Cis-acting factors of DSB formation in the PAR

**Fig. 4:**
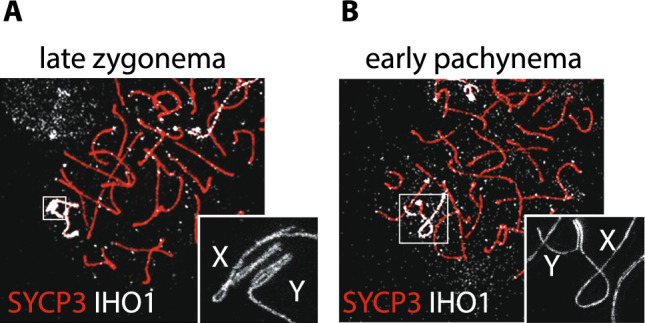
Ultrastructure of the PAR before and after synapsis. **A** Spermatocyte chromosome spread stained with SYCP3 and IHO1, which allows for rapid identification of sex chromosomes [71]. Splitting of the X-PAR and Y-PAR in unsynapsed chromosomes is visible through Stimulated Emission Depletion (STED) super-resolution microscopy imaging (enlargement). **B** Splitting of PARs is lost following XY synapsis

In addition to the presence of mo2-minisatellites (see above and [62]), recent studies have shown that mPAR chromatin in spermatocytes forms relatively short loops on a long axis, compared to autosomes [27]. According to the tethering model [105, 106], this conformation is acquired to increase loops density, favoring DSB formation in the PAR [27]. Using high-resolution structured illumination microscopy, Acquaviva et al. demonstrated that in mice, PAR and telomeres of chromosomes with mo-2 minisatellite repetitive sequences undergo dynamic remodeling [62]. Specifically, the PAR axis elongates, and chromatin loops shorten, as cells progress from leptonema to zygonema. Moreover, as cells approach the late zygotene stage, the sister chromatids of X-PAR and Y-PAR undergo separation (splitting) (Fig. 4A).

These ultrastructural changes are closely correlated with the accumulation of RMMAI blobs and the formation of DSBs [62]. Similarly, telomeres with mo2-minisatellite sequences also undergo splitting paralleling changes in the PAR [62]. Once synapsis is formed, PAR sister chromatids collapse (Fig. 4B), with successive dissociation of RMMAI proteins, shortening of the axes, and elongation of chromatin loops [62]. Importantly, splitting at the PAR and telomeres with mo2-minisatellite is absent in *Mei4*^*−/−*^ and *Ankrd31*^*−/−*^ mutants that fail aggregation of RMMAI proteins [62]. This confirms that sister chromatids separation is somehow related to the formation of RMMAI protein clusters. Nevertheless, the function of PAR (and mo2 minisatellites enriched telomeres) splitting is not yet clear. It has been suggested that axis separation might suppress ineffective intersister recombination in favour of homologous recombination between chromosomes [62, 112]. An alternative (not mutually exclusive) interpretation is that the axes split to accommodate the RMMAI aggregates [112]. In this regard, we demonstrated that in *Spo11βki*-only mice with defective XY synapsis, splitting of the Y-PAR occurs with a slightly reduced frequency compared to wild type control, although apparent normal assembly of RMMAI proteins. Decreased frequency of Y-PAR splitting parallels that of DSB in the Y-PAR [30]. Therefore, we favor the hypothesis that splitting requires the suppression of inter-sister recombination. In addition, in accordance with the tethering model of formation of DSBs, we also observed that reduced formation of DSB in the Y-PAR at late zygonema, correlates with elongation of PAR loops compared to that of wild type. This underlines the importance of the ultrastructural conformation of PAR in the formation of DSBs [30]. It is interesting that the length of PAR loops varies with mouse genetic background [30]. It is therefore plausible that genetic polymorphisms in genes responsible for the ultrastructural organization of chromatin impacts the efficiency of DSB formation on PAR. Further studies will be necessary to explore these aspects in more detail.

### Genetic factors of CO maturation

**Fig. 5:**
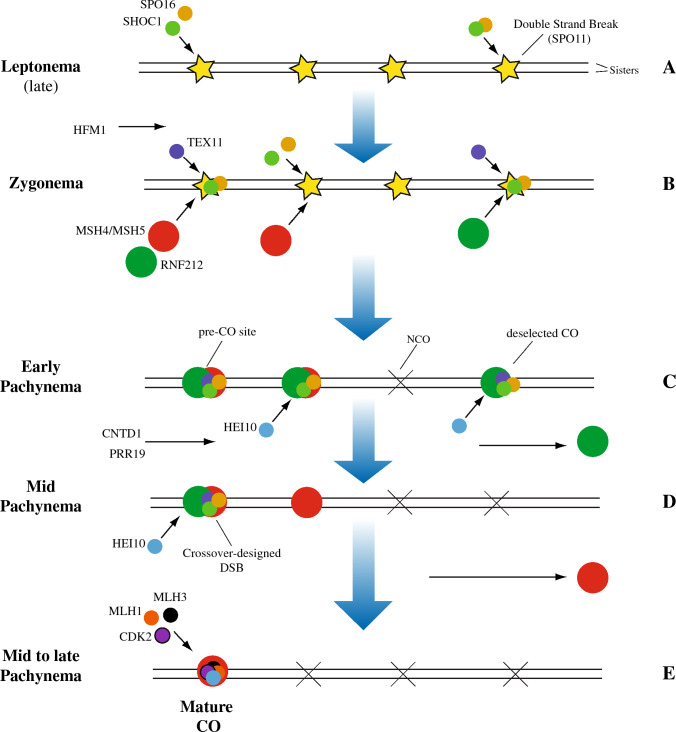
Selection and maturation of COs. Block arrows represent the progression of meiotic recombination. Although four chromatids are present at this stage, only two are shown in the scheme for the sake of simplicity. Recombination proteins assembles at DSB sites as cytologically detectable foci. SPO16 foci are located on chromosome axes, most at synapsed regions; the number of foci increase progressively from leptonema to zygonema and slightly decreases at pachynema. The SPO16 binding partners SHOC1 and TEX11 form foci, which number peaks at zygonema and decreases as the cell progresses to pachynema. MSH4/MSH5 MutSγ proteins also localize at early synapsed regions in zygonema after the turnover of ssDNA binding proteins by HFM1. SPO16, SHOC1 and TEX11 co-localizes only with selected MutSγ positive sites at zygonema, while are lost by late pachynema, when few MSH4/MSH5 persist at designated CO sites. The RNF212 foci number increase as synapsis occurs, up to early pachynema, and subsequently decrease in number to disappear at late pachynema. In mid-pachynema, only one or two foci of RNF212 remain per synaptonemal complex and colocalize with selected MSH4-positive TEX11-positive foci and MutLγ proteins (MLH1 and MLH3) at mature CO sites. CNTD1 and PRR19 narrows down MutSγ-positive and RNF212-positive pre-CO sites with successive loading of HEI10. HEI10 is required for post-synapsis turnover of RNF212. Unlike RNF212, foci of HEI10 are rarely detected along nascent synaptonemal complexes during zygonema, while foci appear by early pachynema, pick at mid-pachynema, and decreases at late-pachynema, when RNF212 foci are already lost, colocalizing with MutLγ proteins at mature CO sites. CO crossover, NCO non-crossover

A DSB formation is the prerequisite but not the guarantee for a CO, which may form only after appropriate processing of DSBs. SPO11-mediated cleavage results in single-strand DNA overhangs that are subsequently coated by various recombination proteins that assembles onto chromosome axes as cytologically visible foci, including the strand exchange factors DMC1 and RAD51 [113–115]. Processing of DSBs allows for homology search, which in turn promotes homology pairing, synapsis, and DSB repair [116]. Repair of DSBs leads to the formation of COs or NCOs [1, 17]. At chromosome scale, the probability of receiving DSBs and resolving them as a CO is negatively correlated with the chromosome size [61]; moreover, the spacing between COs is regulated by a process called COs interference, which causes them be less spatially close than would be expected in a random distribution [1, 56, 117]. COs, subjected to interference are referred to as “Type I”, representing the major fraction (ranging between 90% and 95% in the mouse) of all COs [118]. A minor fraction of COs, not subjected to interference, also forms [1]. These are referred to as “Type II”, which involves structural specific endonucleases such as MUS81 [119]. On average, of all DSBs introduced into the genome only ~ 10–25% is converted into a Type I CO in mammals [16]. Designation of DSBs toward a Type I CO fate requires stabilization of specific DNA intermediates during HR-mediated repair, by pro-CO factors (i.e. directing DSB processing towards a CO fate), collectively known in budding yeast *S. cerevisiae* as ZMM proteins (an acronym of Zip1-4, Msh4-5, Mer3, Spo16). The orthologs and homologous of ZMM proteins in mammalian are SYCP1 (budding yeast Zip1), SHOC1/MZIP2 (ortholog of Zip2), RNF212 (ortholog of Zip3), TEX11 (ortholog of Zip4), MSH4, MSH5, HFM1 (ortholog of Mer3) [118], SPO16 [120]. One additional factor is the Human Enhancer of Invasion-10 (HEI10) (also known as CCNB1 interacting protein 1 [CCNB1IP1] in human), a RNF212 paralog with domain similarity, identified by mouse forward genetic screen [121]. Most of recombination intermediates (namely D-loop structures) stabilized by ZMMs are processed as COs in budding yeast. However, in mammals, ZMM foci outnumber the COs, indicating that DSB intermediates bound by some ZMM proteins at pre-CO sites (i.e. early selected but not yet designated COs) can still be unselected and resolved as NCOs [118], while few DSBs will become COs (designated-CO sites). In mammals, maturation of recombination intermediates toward a COs fate is established progressively, through successive assembly and dissociation of ZMM sub-complexes which partially colocalize with recombination foci defined by RAD51 and DMC1 (single-stranded DNA binding-proteins marking DSBs) [118]. In yeast the ZMM proteins Zip2-Zip4-Spo16 form a stable subcomplex (ZZS) with pro-CO activity [118]. This function and mutual proteins interaction is conserved in mammals, as shown by their mutual co-immunoprecipitation and reduced formation of COs and chiasmata in *Shoc1*^*−/−*^ [122], *Tex11*^*−/−*^ [123] and *Spo16*^*−/−*^ [120] knockout mice, and association of ZZS [124–127] and other ZMM genes deleterious variants [128] with infertility in humans. SHOC1 co-localizes strongly with DMC1 at leptonema and zygonema, indicating a function in stabilizing the early recombination intermediates [122] (Fig. 5A), while co-localization with TEX11 is low at leptonema to early zygonema and increases by late zygonema/early pachynema [122] (Fig. 5B). MSH4-5 MutSγ proteins localize as foci at multiple sites in early synapsed regions (Fig. 5B) at most but not all DSBs, with foci number peaking at zygonema and decreasing at early and late pachynema [51, 122, 129]. This indicates a function in the commitment of a reduced pool of DSBs (i.e. pre-CO sites) toward a CO fate, as demonstrated in yeast [130]. Accumulation of a normal number of MSH4 foci requires HFM1 (helicases for meiosis 1). In the absence of HFM1, normal turnover of earlier recombination intermediates (i.e. RAD51) is impeded, indicating insufficient processing of DSBs [131], causing infertility [132]. Mouse RNF212 and HEI10 that function as small ubiquitin-like modifier (SUMO) and ubiquitin E3 ligases respectively, establish an early differentiation between CO/NCO sites. RNF212 is inferred to promote selective stabilization at a defined intermediate step, of a minority of TEX11 and MSH4-MSH5 bound recombination intermediates [16] beyond early pachynema (Fig. 5C, D), as indicated by their limited co-localization onto axis [16, 133], designating CO sites. The function of RNF212 is conserved in human, as indicated by genome-wide recombination rate changes and meiotic arrest associated with *RNF212* sequence variants [134, 135]. The Cyclin-like cyclin N-Terminal Domain Containing 1 (CNTD1) and its binding partner Proline Rich 19 (PRR19) are the keys to narrow down pre-CO sites marked by MutSγ and RNF212, allowing successive loading of HEI10 [136, 137]. HEI10 is required for post-synapsis turnover of RNF212 and MutSγ co-complexes that culminates in its selective retention at designated CO sites [138] (Fig. 5D, E) and loading of MutLγ factors (MLH3, MLH1) and Cyclin-dependent Kinase-2 (CDK2), with consequent formation of COs [1, 16, 133, 138–140] (Fig. 5E).

#### CO formation in the PAR

In mice and humans DSBs not only form more frequently in the PAR compared with autosomes but are also more likely to be processed toward a CO fate than autosomes [18, 27, 86]. It is estimated that in mouse there is ~ 570-fold higher CO density in the PAR compared to genome average, which translates to a ~ fivefold higher yield of COs per DSB compared to autosomes [61]. Therefore, specific mechanisms are expected to be in place to stabilize CO intermediates toward a CO fate. An important contribution to the formation of the obligatory CO in the PAR comes from ATM (Ataxia Telangiectasia Mutated). *Atm* null spermatocytes are defective in forming the obligate CO on the sex chromosomes [141]. This is in striking contrast to autosomes where the total number of DSBs onto autosomes (and non-homologous portion of sex chromosomes) and COs increases compared to the control [61, 141, 142]. Thus, ATM upgrades DSBs to a CO fate in the PAR, while constraining DSB formation and CO numbers in other chromosome regions. To date, the molecular mechanisms behind such regulatory control are largely unknown. ZMM proteins are certainly necessary for the maturation of DSB in the PAR, as demonstrated by the immunolocalization of MSH4 [140]; in addition, cytological observations show that XY synapsis fails with high frequency in the absence of *Hfm1*, indicating that, like in autosomes, HFM1 is required with MSH4 to initiate or maintain stable early recombination intermediates [131]. RNF212 also localizes at the PAR at early pachynema [133, 138] and is a dosage–sensitive regulator of XY synapsis in mice, likely stabilizing the nascent CO intermediates between PARs [16]. On the contrary, although HEI10 is also a dosage-sensitive regulator of CO formation in mice, no specific defect in HEI10 foci formation, CO maturation or maintenance of stable synapsis between XY chromosomes was reported in heterozygous mice mutants [138]. This could indicate that the stability and maturation of CO intermediates in the PAR is likely not dependent on the HEI10 function.

In addition to the above, over the past 4 years, new genes with an impact on CO maturation have been identified, mainly by sequencing of the human genomes of patients categorized according to their fertility. Some gene functions have been shown to impact mainly recombination between male sex chromosomes, in some cases with no or little impact on autosomal recombination. These genes are described below.

- Genes upgrading DSB formation in the PAR
  *hnRNPH1*: Coordinated regulation of alternative pre-mRNA splicing is essential for germ cell development. An RNA binding protein that has recently been found to play a key role in meiosis is the heterogeneous nuclear riboprotein (hnRNP) hnRNPH1, which absence in germ cells causes male and female sterility, due to altered gene expression and alternative splicing [65]. The lack of hnRNPH1 expression in spermatocytes compromises alternative splicing of genes related to meiosis including *SPO11*, by maintaining SPO11β expression at high level in 18 days post-partum testes, to detriment of the alternative transcript encoding for the SPO11α isoform [65]. This result agrees with a previous report that identified hnRNPH1 as a key regulator of *Spo11α* splicing in mouse spermatocytes [64]. Given that the PAR physiologically receives DSBs with a higher frequency than typical autosome segments [27, 61], and concomitant expression of SPO11β and SPO11α is the key for efficient formation of DSBs in the PAR [30], this is expected to have an impact on the initiation of XY recombination. Accordingly, the authors show that *hnRNPH1*-deficient mice display a tenfold increase of XY asynapsis compared to controls [65]. Furthermore, considering the ability of hnRNPH1 to interact with the splicing factors PTBP2 and SRSF3 in the testes [65], the authors also show that the *Spo11* gene is regulated at the splicing level by PTBP2 and SRSF3c, which are recruited by hpRNPH1 [65].
- Genes upgrading the PAR CO program
  *USP26*: In humans, meiotic XY missegregation can lead to KS offspring. However, to what extent genetic predisposes to paternal sex chromosome aneuploidy has remained long elusive. Liu et al. have demonstrated that deleterious mutations in the *USP26* (ubiquitin-specific protease 26) gene increase the risk of fathering a KS progeny [143]. The study identified *USP26* using a whole-exome sequencing (WES) in a cohort of KS patients, as well as KS family trios. By deleting *Usp26* in mice, they demonstrated that USP26 de-ubiquitinate the ZZS protein TEX11, increasing its expression. *Tex11* mutations have been often associated with male fertility defects [126, 144, 145], and its deletion in mice delayed resolution of DSB intermediates and decreased of CO numbers, with most spermatocytes arresting at pachynema [123, 143]. However, USP26 has several other substrates, including the androgen receptor. Therefore, *Usp26* mutations are expected to impact spermatogenesis via multiple mechanisms [143, 146]. Importantly, some residual spermatogenesis still exists in *Usp26*-deficient mice; spermatozoa are aneuploid for the XY chromosomes [143], and sometime mice sired an XXY offspring [143], demonstrating USP26 potential function in the etiopathogenesis of KS.
  *RAD51AP2*: RAD51-associated protein 2 is a meiosis-specific gene whose frameshift truncating mutations have been identified by WES, in patients with non-obstructive azoospermia. The modeling and phenotypic characterization of mutations in mice have shown that the lack of *Rad51ap2* expression has no impact on processing of DSBs and synapsis of non-sex chromosomes, and although XY chromosomes synapse normally at the PAR in early and mid-pachynema, they separate precociously at late pachynema, before the completion of recombination [147]. Specifically, in the absence of RAD51AP2, the intermediate recombination markers MSH4 and TEX11 vanish precociously from the PAR (but not from autosomes). This demonstrates that RAD51AP2 stabilizes PAR recombination intermediates, playing a key role in maturing the obligatory CO in this chromosomal region. Importantly, the authors also show that RAD51AP2 co-immunoprecipitates with RAD51 (not with DMC1) and interact through the C-terminus of RAD51AP2. Consequently, any mutations occurring in the interaction domains of RAD51AP2 and/or RAD51 within the human genome could potentially have a negative impact on XY recombination. Further WES studies in individuals with KS or a different cohort of non-obstructive azoospermia patients are expected to uncover new variants of these genes, deleterious to recombination in the PAR.
  *M1AP*: Meiosis 1 Arresting Protein is a vertebrate protein expressed only in female and male germ cells. Its mutation has been found to be associated with infertility both in men and in male mice [148–151], however, its molecular structure has remained unidentified. Recently Li et al. [151] modelled the *M1ap* c.1074+2T>C splicing mutation, equivalent to that found in patients with severe oligozoospermia in mice. The mutation causes an inactivating premature protein truncation, causing reduction of both CO formation and chromosome synapses in spermatocytes, particularly between XY chromosomes. Mechanistically, it has been demonstrated that M1AP form discrete foci on the chromosome axes of spermatocytes and that it interacts with the components of the ZZS complex SHOC1, TEX11 and SPO16, colocalizing with the foci TEX11 in a SPO16-dependent manner. Ablation of *M1ap* in mice reduces the recruitment of TEX11 without altering SHCO1 localization, thus altering the stability of early recombination intermediates that mostly affect recombination at the PAR [143, 151].
  *RNF212B*: The ring Finger Protein 212B is a gene whose protein product is characterized by a ring finger domain commonly associated with E3 ubiquitin ligase activity. Serving as the closest paralog to RNF212, RNF212B shares functional significance in controlling recombination rate in mammals [134, 152, 153]. In a recent whole-exome sequency study in patients with severe male infertility Gershoni et al. [154] brought to light a pathogenic variant of the *RNF212B* gene causing a substitution at position 448 (C448T), that results in the conversion of the arginine-150 codon to a premature stop codon. This alteration predicts a truncation of the C-terminal half of the protein. The patients carrying the homozygous *RNF212B*^*C448T*^ variant suffer of a severe chromosome nondisjunction defect in sperm cells, especially of the sex chromosomes [154]. This observation suggests that *RNF212B* has a functional role in the processing of DSBs formed in the PAR, although not strictly specific.
  *ATF7IP2*: The Activating transcription factor 7 interacting protein 2, also called PMS2/MCAF2, is encoded by a gene preferentially expressed in the gonads, especially during the meiosis stage. The null mutation in *Atf7ip2* causes male sterility, predominantly due to defective sex chromosome synapsis failure [155]. Shao et al. have reported that in the absence of ATF7IP2, the length of the chromosome axis increases in autosomes and in the PAR. This correlates with ~ 10% increase of CO frequency in autosomes, while PAR loses the obligatory CO [155]. They ruled out a defect in the occurrence of DSBs at the PAR, while the localization of the ZMM proteins stabilizer RNF212 [16] was impaired, as well that of MSH4. Therefore, the XY CO defect is probably caused by the instability of the recombination intermediates processed by MSH4 and RNF212. Therefore, ATF7IP2 seems to be a protein factor that boosts DSB to CO maturation in the PAR. More recently, a new *Atf7ip2* null model was generated with the same genetic background, which shows no XY synapse defects [156]. The two models differ for the mutated exons (exons 3–6 deletion [155] and 17 bp exon 4 frameshift deletion [156]), which presumably causes the phenotypic difference. ATF7IP2 interacts with the H3K9 histone-lysine *N*-methyltransferase SETB1, to regulate SETB1 retention in the nucleus and H3K9 trimethylation of the chromatin of sex chromosomes [155, 156]. This function is required to regulate MSCI [155, 156], needed for successful spermatogenesis (see [48] and references therein).

## Checkpoint mechanisms of selection of XY aneuploid germ cells

XY aneuploidy (but not autosomal aneuploidy) in human sperm increases with age, often in the face of even modest meiotic perturbations, with the risk of fathering a child with KS [157–159]. Based on recent experimental findings, the association between paternal age and XY aneuploidy lies, at least in part, in the weakening of Spindle Assembly Checkpoint (SAC) mechanisms that eliminate metaphase spermatocytes with misaligned chromosomes [160]. A limiting step in the study of SAC proteins during the meiotic progression of multicellular organisms is the identification of viable alleles since these proteins are essential for proper embryonic development. Therefore, only few studies have addressed whether the SAC mechanism is functional during meiotic progression in vivo, by genetically reducing the dosage of SAC protein [161]. Faisal et al., demonstrated that reduced MAD2 level dampens the apoptotic response in a mouse model with a high frequency of nonexchange XY chromosomes, with consequent generation of sperm aneuploid for the sex chromosomes [29]. Other genes whose heterozygosity led to aneuploid splenocytes (*Bub3*, *Rae1*, *Bub3*/*Rae1* double heterozygotes and *Rae1*/*Nup98* double heterozygotes) did not show such effect in spermatocytes [162], underlining the specificity of *Mad2* function in these cells. In *Usp26*^*−/−*^ mice XY diploid sperm (and more rarely sex chromosomes-nulliploid sperms) were produced only by aged 6-month-old *Usp26*^*−/−*^ mice, when the level of SAC proteins (namely: MAD2, BUBR1, PLK1) was reduced; and aged mice sometime sired XXY offspring [143]. Therefore, it is likely that a combination between inefficient XY pairing and less stringent SAC surveillance allows XY sperm aneuploidy [163].

## Concluding remarks

Male sex chromosome aneuploidy in sperm is a risk factor for infertility, subfertility and generation of a progeny with KS and TS. However, whether and to what extent genetic factors may predispose to XY aneuploidy has long remained elusive. In recent years important progress in understanding the genetics behind XY recombination failure, with the identification of genes whose products have a specific or predominant function in recombination in the PAR. The experimental approaches have varied, from basic research in the field of meiosis to those based on DNA sequencing from infertile patients or patients with KS and their parents. The genes for mutations impacting XY recombination range from those involved in the formation of DSBs and PAR remodeling to those required for the processing of DSBs and the apoptotic selection of cells with an unbalanced number of XY chromosomes. Where gene mutations have been found to be associated with infertility or KS, these mutations were present in a limited pool of affected individuals and parents. This indicates that the catalogue of genes with mutations related to KS and infertility is probably much larger than what we know now. Mutations in individual genes are expected to be genetically unselected and likely to cause XY aneuploidy when in homozygosity and/or expressed in combination with other gene mutations weakening either recombination, chromosome segregation, and/or checkpoint mechanisms efficiency. Therefore, the path to identify genetic risk factors for XY aneuploidy remains a challenge for the future, to be continued.

## Data Availability

All data are available through the cited bibliographic references.
